# Supplementary figures and images for: Enantioseparation and Determination of Penconazole in Rat Plasma by Chiral LC-MS/MS: Application to a Stereoselective Toxicokinetic Study
Source: Molecules. 2020 Jun 28;25(13):2964. doi: 10.3390/molecules25132964 (PMC7411863; doi:10.3390/molecules25132964)

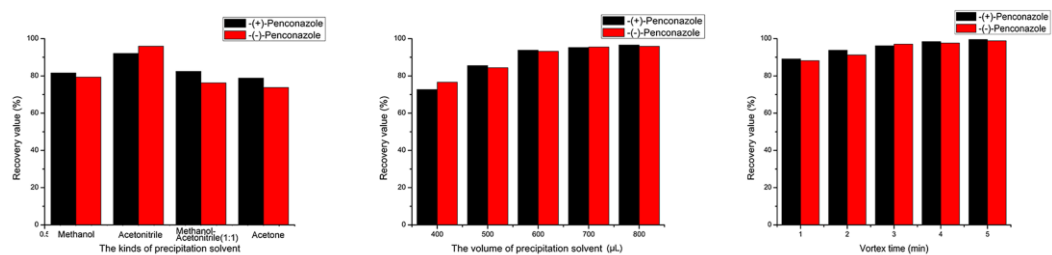

**Figure S1.** The optimization of PPT sample pretreatment method for penconazole enantiomers.

Supplement: Supplementary file 1 [file molecules-25-02964-s001.pdf]
